# POSS Hybrid Robust Biomass IPN Hydrogels with Temperature Responsiveness

**DOI:** 10.3390/polym11030524

**Published:** 2019-03-20

**Authors:** Yi Chen, Yueyun Zhou, Wenyong Liu, Hejie Pi, Guangsheng Zeng

**Affiliations:** 1 Hunan Provincial Key Laboratory of Comprehensive Utilization of Agricultural and Animal Husbandry Waste Resources, College of Urban and Environmental Sciences, Hunan University of Technology, Zhuzhou 412007, China; zyenn@21cn.com (Y.Z.); pihejie@163.com (H.P.); 2 Hunan Provincial Engineering Laboratory of Key Technique of Non-metallic Packaging Waste Resources Utilization, Hunan University of Technology, Zhuzhou 412007, China; lwy@iccas.ac.cn; 3 Hunan Provincial Key Laboratory of Biomass Fiber Functional Materials, Hunan University of Technology, Zhuzhou 412007, China

**Keywords:** octa-ammonium POSS, sodium alginate, hydrogels, temperature responsiveness

## Abstract

In order to improve the performance of traditional sodium alginate (SA) hydrogels cross-linked by Ca^2+^ ions to meet greater application demand, a strategy was designed to structure novel SA-based gels (named OP-PN gels) to achieve both stimulus responsiveness and improved mechanical strength. In this strategy, the SA chains are co-cross-linked by CaCl_2_ and cationic octa-ammonium polyhedral oligomeric silsesquioxane (Oa-POSS) particles as the first network, and an organically cross-linked poly(N-isopropyl acrylamide) (PNIPA) network is introduced into the gels as the second network. Several main results are obtained from the synthesis and characterization of the gels. For OP-PN gels, their properties depend on the content of both uniformly dispersed Oa-POSS and PNIPA network directly. The increased Oa-POSS and PNIPA network content significantly improves both the strength and resilience of gels. Relatively, the increased Oa-POSS is greatly beneficial to the modulus of gels, and the increased PNIPA network is more favorable to advancing the tensile deformation of gels. The gels with hydrophilic PNIPA network exhibit better swelling ability and remarkable temperature responsiveness, and their volume phase transition temperature can be adjusted by altering the content of Oa-POSS. The deswelling rate of gels increases gradually with the increase of POSS content due to the hydrophobic Si–O skeleton of POSS. Moreover, the enhanced drug loading and sustained release ability of the target drug bovine serum albumin displays great potential for this hybrid gel in the biomedical field.

## 1. Introduction

Hydrogels are physically or chemically cross-linked three-dimensional hydrophilic polymeric networks capable of absorbing large amounts of water (or biological fluids) and swelling. These soft materials have similar properties to human tissue and show responsiveness to special stimuli, attracting growing interest in recent years due to their potential application in tissue engineering [1,2], soft robotics [3,4], biosensing [5,6], flexible displays [7,8], and drug delivery [9,10]. In the synthesis of hydrogels, both natural and synthetic polymers are used based on their respective advantages. Natural polymers, including cellulose, chitin, chitosan, sodium alginate, gelatin, and pectin, have received more attention for their low cytotoxicity as well as their biocompatibility and biodegradability, which are advantageous in the biomedical field [11,12,13]. Among natural polymers, sodium alginate (SA)—one of the most abundant polysaccharides, which is extracted from brown seaweed—is very promising and has been widely exploited in designing oral delivery of protein or peptide drugs [14,15,16]. The mucoadhesive property of alginate has also made it a favored formulation excipient in the pharmaceutical industry [17]. More interestingly, it can undergo sol–gel change under special conditions by physical cross-linking. SA undergoes supramolecular assembly in acid at pH below 3, forming acid gel, or can form ionotropic physical gel by cooperative binding with multivalent cations, typically Ca^2+^ [18,19]. SA hydrogel has intrinsic biological advantages. For example, SA hydrogels contain carboxylate anions on their hydrophilic surface similar to the characteristics of cell surfaces in vertebrates, which could minimize the effect on immunity, thus advancing biocompatibility [20,21]. Moreover, because of their swelling ability, robust network, fast forming ability, and structural similarity to macromolecular-based components in natural tissue, SA hydrogels have been used as sustained drug delivery systems for active biomacromolecules, as biomimetic extracellular matrices for cell attachment in tissue engineering applications, as biomaterials for protein and cell encapsulation, and as raw material for 3D printing [22,23,24].

Though SA-based hydrogels have achieved great advancements in fundamental and laboratory research, they are still far from being used in industrial or clinical applications, since many challenges still exist. First, their mechanical properties are still poorer than those of synthetic polymer gels, and this deficiency limits their application in some areas such as cell scaffold, which needs enough load capacity to allow cell proliferation. Second, the absence of stimulus responsiveness makes it impossible for gels to achieve controllable drug release under special conditions, which is important in in vivo drug delivery. Third, the new functionality of alginate gels still needs to be exploited and developed in order to meet the demands of complex therapy.

To further improve the mechanical properties and introduce stimulus responsiveness into SA gels, in this work, new nanocomposite SA hydrogels with an interpenetrating polymer network (IPN) structure were designed. Considering that the Ca^2+^ ions cross-link SA through ion exchange, another water-soluble cationic nanoparticle, octa-ammonium polyhedral oligomeric silsesquioxane (Oa-POSS), was chosen to use as a co-cross-linker together with CaCl_2_. POSS has a compact hybrid structure with an inorganic core made up of silicon and oxygen and eight corner arm groups: g[RSiO3/2]n (with n = 8, 10, and 12); R is H, alkyl, alkylene, aryl, aromatic alkylene, or their derivative. The corner arms are reactive or inertial functional groups, which endow POSS with diversified functionality and reactivity [25,26,27]. According to previous research, introducing POSS particles into polymer-based materials and gels could improve the mechanical properties or endow the matrix with special functions. For example, polyethylene glycol (PEG)-POSS multiblock polyurethane hydrogels have higher stiffness than normal polyurethane hydrogels, in which the POSS nanocrystals serve as physical cross-linking points [28]. Poly(2-hydroxyethyl methacrylate) (PHEMA) and poly(ethylene glycol) dimethacrylate/poly(ethylene glycol) monomethacrylate (PEGDM/PEGMM) hydrogels cross-linked by methacryloxy-multifunctionalized POSS showed improved surface properties, swelling ability, and mechanical properties in the swollen state [29]. POSS particles were also used to prepare poly(N-isopropyl acrylamide) (PNIPA) gels as cross-linker or additive. Zeng prepared a series of rapid thermoresponsive POSS-containing PNIPA hydrogels by using POSS with various long flexible chains, and obvious hydrophobic nanodomains in gels were observed by atomic force micrography [30]. Octa (propylglycidyl ether)-POSS, mercapto-POSS, and octavinyl-POSS were also used in PNIPA gels to improve the deswelling rate and mechanical properties [31,32,33]. Moreover, POSS was proven to have good biocompatibility, which is more promising for use as a biomedical material [34,35]. The IPN structure was applied here to introduce responsiveness and further improve the properties. Previously, many IPN gels were designed and achieved excellent mechanical properties due to their special matched network structure and strong network entanglement for stress dispersion [36]. For example, Gong and co-workers developed IPN hydrogels consisting of poly(2-acrylamido-2-methylpropane sulfonic acid) (PAMPS) for the first network and polyacrylamide (PAAm) for the second network, and the gels showed extremely high mechanical strength greatly beyond the original strength of the respective networks [37]. Besides, many functions of the respective network are brought into the new IPN gels or combined to achieve complementarity [38,39].

Based on the above strategy, novel hydrogels were fabricated by combining two different networks: the first one is the SA co-cross-linked by CaCl_2_ and Oa-POSS collectively, and the second network is the organically cross-linked PNIPA. The structure of this gel was comprehensively investigated by Fourier transform infrared spectroscopy (FTIR), X-ray diffraction (XRD), differential scanning calorimetry (DSC), thermal gravimetric analysis (TGA), scanning electron microscopy (SEM), and optical microscopy (OM), and the properties of gels—including mechanical properties, swelling, and deswelling behavior—were measured. Moreover, to determine its potential in drug release, bovine serum albumin (BSA) was used as the target drug to analyze the loading and releasing ability of the gels.

## 2. Materials and Methods

### 2.1. Materials

NIPA monomer, potassium peroxodisulfate (KPS), N,N’-methylenebisacrylamide (BIS), and N, N, N, N-tetramethylethylenediamine (TEMED) were provided by TCI Co., Tokyo, Japan. NIPA was purified by recrystallization from a toluene/n-hexane mixture (2/1 *w*/*w*) and dried under vacuum at 40 °C. Sodium alginate (SA) and calcium chloride and were purchased from Aladdin Co., Shanghai, China, and used without further purification. The viscosity of SA (1 wt %, 20 °C) is 400 ± 50 mPa.s. Oa-POSS was provided by Hybrid Plastics Co., Irvine, California, USA. BSA was purchased from J&K Chemical Co., Shanghai, China.

### 2.2. Methods

#### 2.2.1. Synthesis of OPn-PNm Gels

The POSS hybrid SA/PNIPA IPN gels (OPn-PNm gels) were prepared by a two-step reaction, and the process of synthesis was as follows: A given amount of sodium alginate, KPS, and TEMED was mixed with deionized water in a flask and stirred by a magnetic stirrer for 1 h at room temperature to get a homogeneous solution, then a mixed aqueous solution with monomer NIPA and cross-linker BIS was added to the sodium alginate solution. The solution was stirred quickly for 2 min and then transferred to a mold with certain shape and sealed. Free-radical polymerization was carried out at 20 °C for 24 h to form the first network gels. In the gels, the concentration of sodium alginate was fixed at 2%; the mass ratio of water, NIPA, initiator KPS, and catalyzer TEMED was 1000:100:1.02:0.755; and the molar ratio of BIS and NIPA was fixed at 4%. Then, the formed gels were immersed into supersonic 3 wt % CaCl_2_ and Oa-POSS aqueous solution at 20 °C for 48 h to form the second network inside the gels. Finally, the prepared gels were swollen and deswollen repeatedly in deionized water to remove the unreacted monomer as purification.

In this research, the resulting prepared gels are expressed as OPn-PNm gels, using a simplified numerical value of Oa-POSS content and the ratio of both networks. For OPn-PNm gels, n is defined as 1/4 × mass percentage of Oa-POSS in total CaCl_2_ and Oa-POSS for a simple expression, and m corresponds to the mole ratio of NIPA to a single sodium alginate molecule. For example, OP3-PN3 gel means that the mass of Oa-POSS in aqueous solution is 12% of all CaCl_2_ and Oa-POSS mass, the mole ratio of NIPA is 3 times that of SA. In this research, n and m are equal to or less than 5 to guarantee the homogeneity of gels.

#### 2.2.2. Characterization of OPn-PNm Gels

FTIR spectra of the gels were taken under ambient conditions by a Nicolet 380 FT-IR spectrometer (Thermo Fisher Scientific Inc., Waltham, MA, USA) with milled, dried, unpurified hydrogels by the conventional KBr disk tablet method.

XRD patterns were conducted on a D8 advance X-ray diffractometer (Bruker AXS Inc., Karlsruhe, Germany) using Cu Kα radiation at 40 kV. The diffraction data were collected with a 2θ angle in the range of 2° to 60° at a scanning rate of 5/min.

TGA was analyzed by a Q50 thermogravimetric analyzer (TA Instruments Inc., Newcastle, DE, USA) at a heating rate of 20 °C/min in the range of 20 °C to 600 °C under a nitrogen atmosphere, with a flow rate of nitrogen of 30 mL/min.

DSC was used to determine the volume phase transition temperature (VPTT) of the samples by using a Q20-Series differential scanning calorimeter (TA Instruments Inc., Newcastle, DE, USA). All samples used in DSC measurement were immersed in deionized water at room temperature and allowed to swell for at least 72 h to reach the equilibrium state. The calorimetric analysis was performed from 20 °C to 60 °C at a heating rate of 1 °C/min under a dry nitrogen atmosphere, and the flow rate of nitrogen was 20 mL/min.

The morphology of the gels’ network structure was observed by a S3000-N low-vacuum scanning electron microscope (Hitachi High Technologies America Inc., Chandler, AZ, USA). Each sample for observation was rapidly frozen and subsequently freeze-dried for 48 h under vacuum at −60 °C. The dispersion of Oa-POSS in the gels was also observed by SEM, and the samples for observation were etched by 25 wt % hydrofluoric acid (HF) solution to dissolve and eliminate the inorganic particles and then dried under vacuum at 30 °C. The surface morphology of gels was observed by a LV100POL/50iPOL polarizing microscope (Nikon Inc., Tokyo, Japan).

#### 2.2.3. Mechanical Measurements

Tensile mechanical measurements were performed on gels of the same size, 10 × 3 × 80 mm (width × thickness × length), using a UTM6000 universal mechanical tester (Suns Inc., Shenzhen, China). The sample length between the jaws was 35 mm and the crosshead speed was 10 mm/min. An initial cross-section of 20 mm^2^ was used to calculate the tensile strength and modulus, and the tensile strain was taken as the length change relative to the initial length of the sample. The tensile modulus was calculated from the increase in load detected between elongation of 10% and 45%. Compression tests were carried out using samples of the same size, 10 × 10 × 10 mm (width × thickness × length), on the UTM6000 universal mechanical tester (Suns Inc., Shenzhen, China). The compression property of all gels was obtained under the following conditions: compression speed 5 mm/min and compression distance 8 mm (80% strain). All gels for testing maintained the same water/polymer ratio (8/1 (*w*/*w*)).

#### 2.2.4. Dynamic Rheological Experiments

The rheological tests were carried out on a stress-controlled AR2000 rheometer (TA company, Newcastle, DE, USA) in dynamic mode at 20 °C. The linear viscoelasticity region was confirmed by dynamic strain sweeps at a constant frequency of 1 rad/s. The storage modulus G’ and loss modulus G” were characterized by dynamic frequency sweeps from 0.1 to 200 rad/s, conducted in fixed strain γ = 1%, which is within the linear regime confirmed by dynamic strain sweeps.

#### 2.2.5. Swelling Experiment

The swelling experiment was performed by immersing the dried gels in water at 20 °C for at least 48 h to reach equilibrium. The weight of the gel was measured after wiping off the excess water on the surface with moistened filter paper. The swelling ratio was calculated from the equation

Swelling ratio (SR) = (Ws − Wd) / Wd(1)

where Ws is the weight of swollen hydrogel and Wd is the dry weight of the hydrogel.

#### 2.2.6. Deswelling Kinetics Experiment

The deswelling kinetics of the hydrogels after a temperature jump from the equilibrated swollen state at 20 °C to hot water at 50 °C were measured after wiping off the excess water on the surface with moistened filter paper. The water retention corresponding to the deswelling ratio was calculated from the equation

Water retention (WR) = (Wt − Wd) / Ws × 100%(2)

where Wt is the weight of swollen hydrogel at a specific time.

#### 2.2.7. Drug Load and Release Experiment

Synthesis of hydrogel–drug conjugate was carried out. In the first step of synthesis, BSA was added to sodium alginate aqueous solution to form a homogeneous solution to achieve imbedding. Aside from this step, the process was consistent with the synthesis of blank gels. The loading percentage of BSA was tested by a difference value between added mass and dissolving mass of BSA in reaction condition (CaCl_2_ and Oa-POSS aqueous solution), BSA concentration was measured by UV spectroscopy (Lambda 950 UV/vis spectrophotometer, Perkin-Elmer Inc., Waltham, MA, USA), and absorbance was monitored at 280 nm [40].

The protein-loaded gels were placed into a 200 mL aqueous solution containing 0.02 w/v % sodium azide as a bacteriostatic agent at 25 °C. At a certain time interval, the gels were removed from the solution and the solution was mixed to homogeneity by a rotary shaker at a speed of 100 rpm. The release content was calculated by the concentration of BSA in the solution, which was measured by UV spectroscopy.

## 3. Results and Discussion

### 3.1. Synthesis and Structure of OPn-PNm Gels

**Figure 1:**
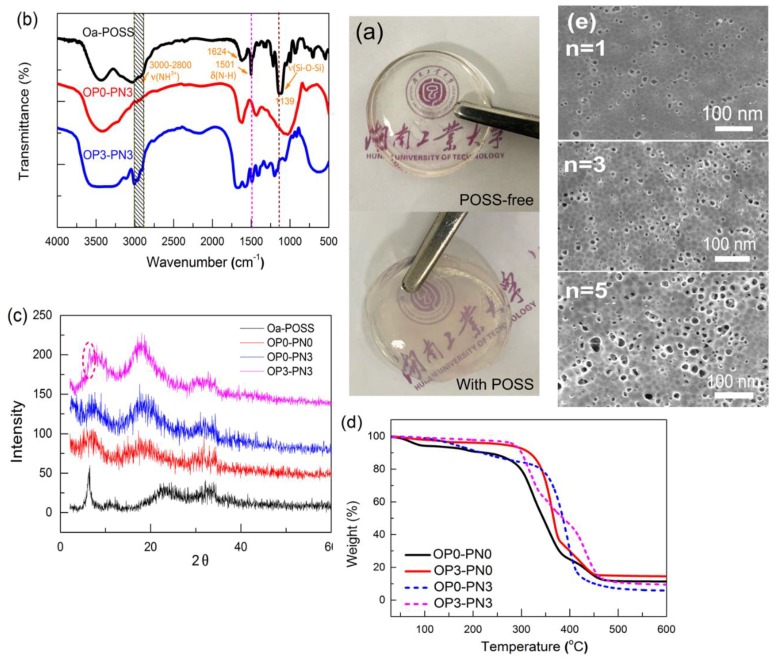
Appearance and structure analysis of OPn-PNm gels: (**a**) photos of interpenetrating polymer network (IPN) gels with and without octa-ammonium polyhedral oligomeric silsesquioxane (Oa-POSS); (**b**) FTIR spectra of Oa-POSS particles and OPn-PNm gels; (**c**) X-ray profiles of Oa-POSS particles and OPn-PNm gels; (**c**) thermogravimetric curves of OPn-PNm gels; (**d**) SEM images of etched OPn-PN3 gels (n = 1, 3, 5).

When it contacted the multivalent cations, the sodium alginate (SA) could form the ionotropic physical gel rapidly by ion exchange. To structure IPN hydrogels based on SA, NIPA was chosen to be cross-linked first to form a precursor gel with SA, and then the SA network was formed inside the precursor gel by immersing the gel into the solution with abundant exchangeable ions. Polymerization yields, evaluated from the weights of dried gels, were nearly 100% in all cases, revealing a complete reaction. Our concern here is the successful introduction of POSS particles and their link type inside the gels, and several characterizations were used to analyze this problem. First, from the appearance of gels (Figure 1a), it could be observed that gels within the range of the synthetic formula (n ≤ 5) stayed transparent without obvious phase separation or precipitation. Relatively, when the content of Oa-POSS becomes higher (n > 6), some degree of heterogeneity appears inside the gels.

Moreover, when adding Oa-POSS aqueous solution to the SA aqueous solution as the single cross-linker, the viscosity of the solution increases quickly, accompanied by a great amount of white flocculates, and the integral forming of gels is unsuccessful, meaning that the Oa-POSS particles could not be used as the single cross-linker to achieve the preparation of SA gels. However, this phenomenon indicates that the ion exchange between Oa-POSS and SA happens in the system. According to the molecular structural formula of Oa-POSS, there are eight arms with monovalent ions in the corner of the Si–O cage; a high concentration of Oa-POSS should bring about an increased charge density, which makes it easier to cause aggregation of SA around the POSS particles instead of uniform cross-linking.

Figure 1b shows the FTIR spectra of the Oa-POSS, OP0-PN3, and OP3-PN3 gels. Comparing these absorption curves, the curve of OP3-PN3 gel contains the same characteristic absorption peaks as the curve of Oa-POSS, in which a broad peak between 2800 and 3000 cm^−1^ corresponding to the stretching vibration of (NH3^+^), 1624; 1501 cm^−1^ corresponding to the bending vibration of (N–H); and 1139 cm^−1^ corresponding to the stretching vibration of (Si–O–Si) could be observed. This feature proves the existence of Oa-POSS inside the gels [41].

Moreover, as shown in the X-ray profiles of Oa-POSS, pure SA, OP0-PN3, and OP3-PN3 gels in Figure 1c, the peak of 100 crystal plane belonging to Oa-POSS particles appears at about 2θ = 5°. From the profile of OP3-PN3 gel, this characteristic peak can still be found but it becomes weaker, indicating the existence of slight crystals of Oa-POSS inside the gel. It was also observed that the diffraction peak at 2θ = 8° belonging to SA was strengthened after adding Oa-POSS, which may be attributed to the tighter combination of SA around the Oa-POSS particles promoting the crystallization of SA.

Thermogravimetric analysis was also used for the gels. Two groups of TG curves belonging to SA and IPN gels with or without Oa-POSS were compared. As shown in Figure 1d, for SA gels (OP0-PN0, OP3-PN0), the TG curve of POSS hybrid gel shifts to the higher temperature more than that of neat gels, meaning there was better thermostability caused by the addition of Oa-POSS. Furthermore, the residual mass of OP3-PN0 gel was higher than that of OP0-PN0 gel. Obviously, the co-cross-linking by Oa-POSS led to a tighter network structure, which is conducive to thermostability, and the Oa-POSS particles had a higher thermolysis residue ratio than other ingredients in the gels, resulting in a high residual mass. A similar phenomenon was found in IPN gels (OP0-PN3, OP3-PN3), with a slight difference. The POSS hybrid gels had better thermostability in the low temperature region, and the temperature corresponding to their maximum decomposition rate was higher than that of neat gels. This may be attributed to a structural feature of POSS hybrid IPN gels, in which a part of PNIPA polymer chains are wrapped by the SA network co-cross-linked by Oa-POSS, and the tighter network causes harder heat transfer, resulting in a higher weight loss temperature. Moreover, higher residue mass was also found in POSS hybrid gels. Compared to the SA gels cross-linked only by CaCl_2_, the gels cross-linked by POSS particles had a higher ratio of solid residue (e.g., if 1 mol of CaCl_2_ or Oa-POSS corresponds to 2 mol SA equally, the percentage of residue mass is 14.7% and 33.1%, respectively; if 1 mol Oa-POSS corresponds to 4 mol SA, the residue mass percentage is 26.7%; the reality may be between these situations). Though the actual situation is complex, the combination type (as cross-linker or pure additive) and the cross-linking degree (1 mol Oa-POSS cross-links 1–8 mol SA) are hard to define, and the increased residue mass could still prove the successful introduction of Oa-POSS particles.

Figure 1e shows the surface morphology of OPn-PN3 gels (n = 1, 3, 5) etched by HF solution to dissolve the POSS particles. As shown in the figure, the holes, corresponding to the dissolved Oa-POSS particles, are dispersed uniformly in the gel, essentially revealing uniform dispersion of Oa-POSS particles inside the gel. The quantity of pores increases with the increased Oa-POSS concentration used in the reaction, indicating that the Oa-POSS content inside the gels directly depends on the Oa-POSS concentration in the reaction process. However, the size of the holes (10–30 nm) is bigger than the original size of Oa-POSS (2–4 nm), especially when the content of Oa-POSS is high, indicating a certain degree of aggregation inside the gels. Based on the above analysis, it can be concluded that the Oa-POSS content is proportional to Oa-POSS concentration in the reaction solution.

### 3.2. Mechanical Properties of OPn-PNm Gels

**Figure 2:**
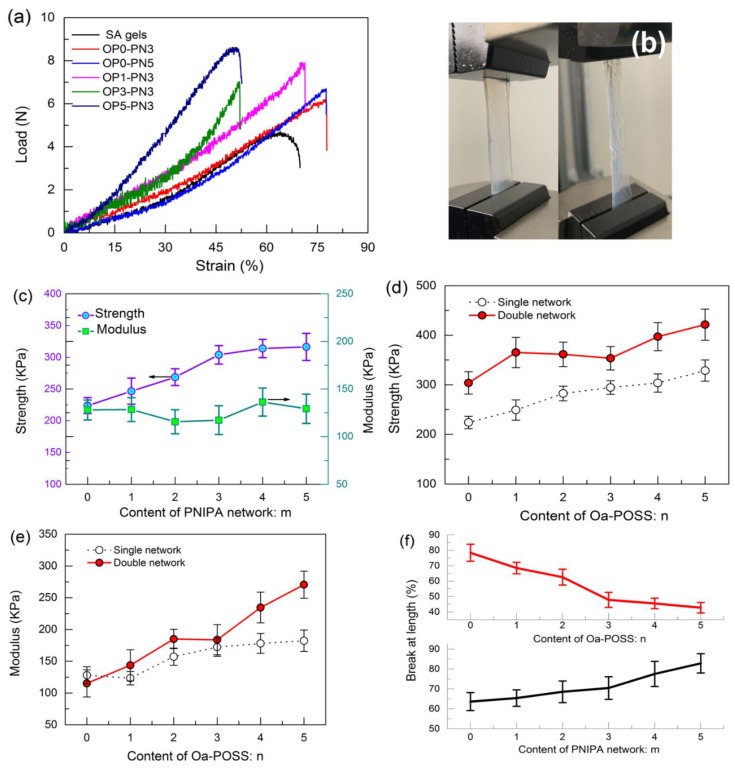
Tensile properties of OPn-PNm gels: (**a**) load–strain curves of gels in tensile tests; (**b**) images of OP3-PN3 gel in tensile tests; (**c**) calculated strength and modulus of OP0-PNm gel; (**d**) calculated strength of OPn-PN0 and OPn-PN3 gels; (**e**) calculated modulus of OPn-PN0 and OPn-PN3 gels; (**f**) elongation at breakage of OP1-PNm and OPn-PN3 gels.

Although pure SA hydrogel is not easy to break under compression and small deformation, its strength and resilience still have room to advance in order to meet application demands. The tensile curves of different OPn-PNm gels are shown in Figure 2a. As shown in the figure, the interpenetrating network structure significantly improves the strength and elongation at breakage of gels. The introduction of Oa-POSS particles could further increase the strength, while it should cause earlier breakage of gels in tensile tests. However, the POSS hybrid IPN gels still could be stretched uniformly, showing ductile deformation (Figure 2b).

From the specific data shown in Figure 2c–e, it can clearly be seen that the IPN gels show higher strength with increased PNIPA network content; the tighter network structure is more beneficial to stress loading, while the modulus increases slightly or even decreases when the PNIPA content is high. Comparing the nature of the two networks inside the gels, the SA network has higher strength than the PNIPA network, revealing rigid characteristics. Therefore, the initial stress should mainly be loaded by the SA network; the PNIPA network only works as a collaborator to share part of the stress. When the content of PNIPA increases and takes on the main role for stress loading, the modulus is inclined to close to the feature of the PNIPA network. Moreover, elongation at breakage of gels increases monotonously with the increased PNIPA network content; obviously, the entanglement of cross-linked polymer is helpful to the deformation of the polymer network.

On the other hand, introducing Oa-POSS truly improves the strength and modulus of both pure SA gels and IPN gels, especially IPN gels. The modulus of OP5-PN3 gels is advanced almost three times that of OP0-PN3 gels. This is ascribed to the overlapped effect of tighter network structure and better stress dispersion by nanoparticles. However, a mass of Oa-POSS should decrease elongation at breakage of gels; the dispersed POSS particles acting as stress concentration points in the gel network lead to increased heterogeneity of the gel, which brings about earlier heterogeneous fracture.

**Figure 3:**
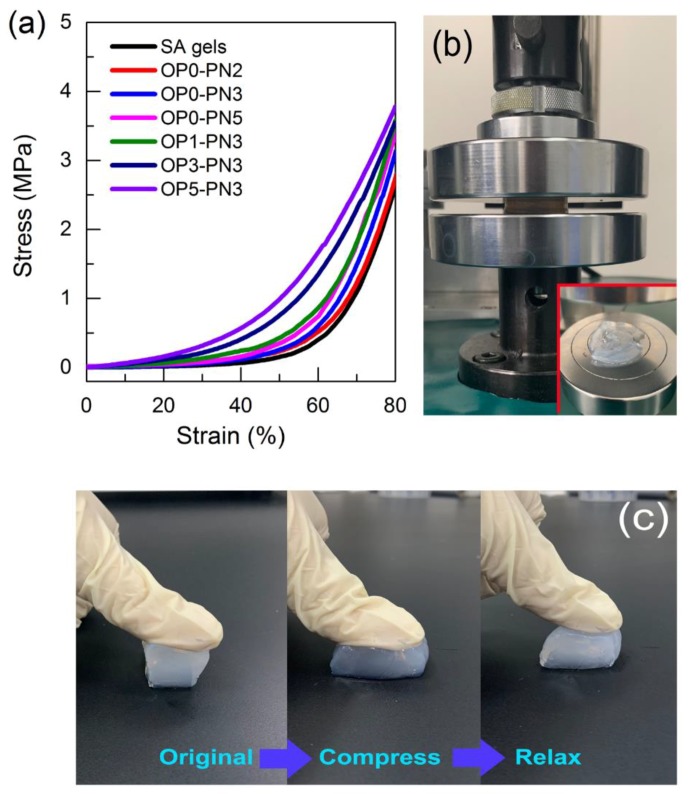
Compression properties of OPn-PNm gels: (**a**) stress–strain curves of sodium alginate (SA) and OPn–PNm gels under compression tests; (**b**) OP3-PN3 gels under 95% strain compression; (**c**) compression and resilience behavior of OP3-PN3 gels.

The compression properties of pure SA gels and OPn-PNm gels were measured by using a uniaxial compression test. As shown in Figure 3, all gels could bear great compression deformation and stay unbroken even under greater than 90% strain (Figure 3b). The corresponding stress–strain curves of OPn-PNm gels with different n and m under compression (up to 80% compression) are shown in Figure 3a. As shown in the figure, the introduction of IPN structure and POSS particles increases the rigidity of the gel network, resulting in higher compression strength. Notably, the gels with Oa-POSS show better resilience under compression (Figure 3c) than SA gels, revealing that the interpenetrating network structure and Oa-POSS endow the polymer network with better elasticity.

### 3.3. Viscoelastic Behavior of OPn-PNm Gels

**Figure 4:**
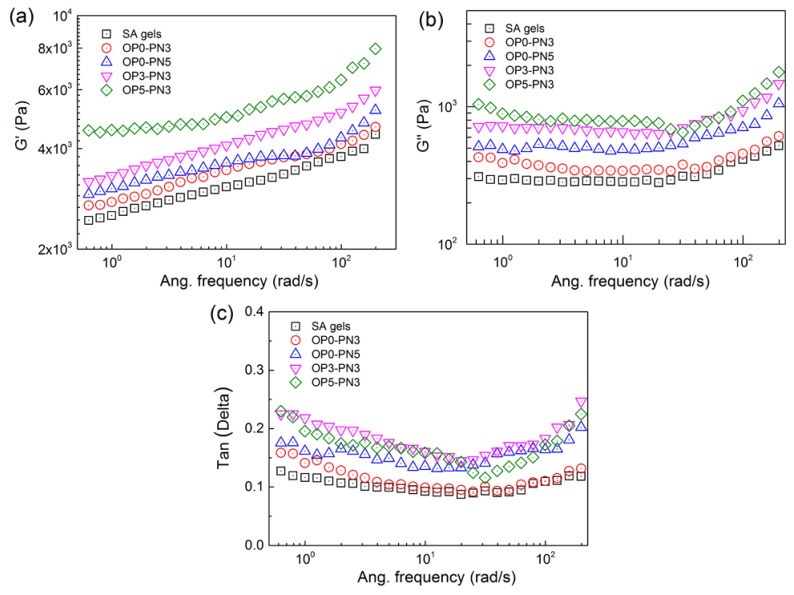
Rheological behavior of SA and OPn-PNm gels measured under a dynamic frequency sweep at fixed strain, γ = 1%, which is in linear viscoelastic region LVE. (**a**,**b**) G’ and G” of gels; (**c**) tan(Delta) of gels.

The structural characteristics of different gels were further confirmed by rheology tests, and several rheological features are shown in Figure 4. First, for all tested hydrogels, the storage modulus (G’) was higher than the loss modulus (G”) over the entire frequency range (0.1 to 200 rad/s), with G’ in the order of 10^3^–10^4^ Pa, indicating elastic solid behavior. Second, G’ increased gradually with the increase of frequency for both SA and OPn-PNm gels, indicating that elasticity increases with increased frequency, while G”, corresponding to the viscidity of the network, was stable under low frequency and increased at high frequency. Third, the IPN structure and increased Oa-POSS content led to higher G’, revealing higher strength and better elasticity, which is consistent with the results of tensile tests. The same was found for the G” evolution of gels, meaning greater network viscidity caused by IPN structure and POSS co-cross-linking. Finally, the evolution of the damping factor (tan(δ)) for all gels with shear frequency is shown in Figure 4c. Totally, the tan(δ) of OPn-PNm gels was higher than that of pure SA gels, revealing a better damping effect due to the IPN structure. Relatively, the effect of Oa-POSS content change on tan(δ) was slight. The introduction of the PNIPA network tightens the network structure of gels, and the interface between the two kinds of polymer chains causes increased viscosity, resulting in greater energy consumption, which could also be proved by the increased tan(δ) of IPN gels at high frequency.

### 3.4. Swelling and Responsiveness Properties of OPn-PNm Gels

**Figure 5:**
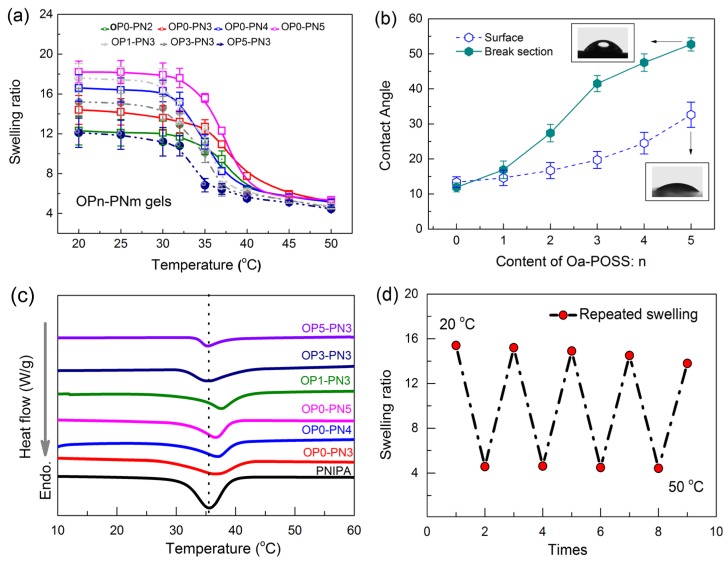
Swelling properties of OPn-PNm gels: (**a**) equilibrium swelling ratio of OPn-PNm in pure water at different temperatures; (**b**) water contact angle of OPn-PN3 gels; (**c**) differential scanning calorimetry (DSC) curves of OP0-PNm and OPn-PN3 gels; (**d**) repeated swelling ability of OP3-PN3 gels. Gels were tested until reaching equilibrium in 20 and 50 °C water alternatively.

**Figure 6:**
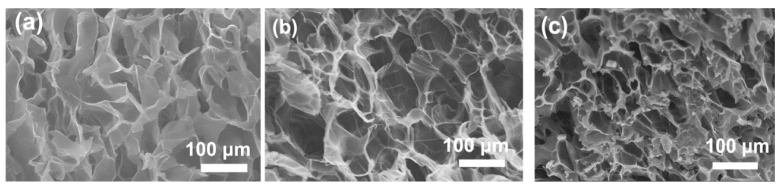
SEM images of SA, OP0-PN3, and OP3-PN3 gels. Network morphology of gels for observation was prepared by freeze-drying. (**a**) SA gels; (**b**) OP0-PN3 gels; (**c**) OP3-PN3 gels.

After introducing the hydrophilic PNIPA network, the swelling ability of gels underwent a remarkable change, and at the same time temperature responsiveness was also brought into the gels. Figure 5a shows the equilibrium swelling ratio of OPn-PNm gels as a function of temperature; it was found that the swelling ratio under low temperature was markedly differentiated. When the content of the PNIPA network increased in the gels, the swelling ability improved gradually; the swelling ratio of OP0-PN5 gels could reach about 18, nearly two times that of pure SA gels. On the contrary, adding Oa-POSS led to a great decrease in swelling ability; the swelling ratio of OP5-PN5 gels decreased to about 10, only half that of OP0-PN5 gels. Although the Oa-POSS is water-soluble due to the existence of ionic groups in the corner of the macromolecule, its Si–O–Si skeleton still exhibits hydrophobic features, which makes it oppose the combination with water. This can be supported by the test results, including water contact angle (CA) on both the surface and break section of gels. As shown in Figure 5b, with increased Oa-POSS in the gels, the CA on the gel surface increased slightly, while the CA on the break section increased significantly. As described in the experimental section, the gels were synthesized and formed in an aqueous environment, and the hydrophilic segment should tend toward the water and build up a more hydrophilic surface. Therefore, the break section is more representative of the true status inside the gels. Moreover, introducing the second network and adding Oa-POSS as the co-cross-linker should change the cross-linking density and style of the gel network. As shown in Figure 6a–c, SEM images of the typical network morphology of pure SA gels, IPN gels, and POSS hybrid IPN gels show that the pore size of IPN gels is smaller than that of single network gels, revealing a tighter network structure. The addition of Oa-POSS intensifies this phenomenon, and a more compact network could be considered as further evidence to prove the role of Oa-POSS as co-cross-linker.

Moreover, according to the swelling characteristics, all gels had a volume phase transition temperature (VPTT) corresponding to the drastic decrease of swelling ratio with increased temperature. Although the lower critical solution temperature (LCST) of PNIPA macromolecule chains is unique, the OPn-PNm gels showed differential VPTT due to the interaction between both networks. Thermoanalysis was used to display the VPTT of gels more directly. Figure 5c shows DSC thermograms of OPn-PNm gels; here, temperature at the maximum point of the endotherm is referred to the VPTT as illustrated in the figure for a better comparison. As we can see, all OPn-PNm gels exhibit a VPTT near 35 °C, which is close to that of pure PNIPA hydrogels. IPN gels without Oa-POSS show higher VPTT, about 37–38 °C, which may be attributed to the rigid SA network impeding the shrinking PNIPA network. With increased Oa-POSS content, the VPTT gradually returns to lower temperature. The reason may be that the hydrophobic cage skeleton of POSS nanoparticles works as a core to promote the shrinking of the network when the phase transformation happens. Another phenomenon should be noted, that the shrinking of gels is incomplete with reserved swelling ratio of about 4–5. Obviously, the rigid and hydrophilic SA network prevents the PNIPA network from completely shrinking. Finally, the repeated swelling ability of OPn-PNm gels was tested by swelling and deswelling the gels in 20 and 50 °C water to reach equilibrium alternatively. The result in Figure 5d shows that the gels have good swelling–deswelling repeatability.

### 3.5. Deswelling Properties of OPn-PNm Gels

The addition of the PNIPA network endows the gels with responsiveness. The response rate of gels, as one of the most important factors that must be considered for the application of thermosensitive hydrogels in many fields, was analyzed by testing the deswelling kinetics.

**Figure 7:**
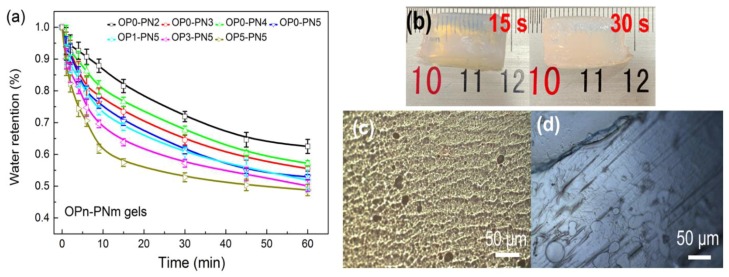
Deswelling properties of OPn-PNm gels: (**a**) water retention of OPn-PNm gels as a function of time in 50 °C water; (**b**) photo of OP5-PN5 gels after being immersed in 50 °C water for 15 s and 30 s; (**c**) optical microscopy (OM) image of OP5-PN5 gel surface in deswelling process; (**d**) OM image of OP0-PN5 gel surface in deswelling process.

Figure 7a shows the water retention change of OPn-PNm hydrogels after a temperature jump from equilibrated swollen state at 20 °C to ultrapure water at 50 °C. It can be clearly seen that the deswelling rate of OPn-PNm gels increases significantly with the increased PINPA network. The deswelling rate of OP0-PN5 gels is close to that of pure PNIPA gels, revealing only slight obstruction from the SA network.

Relatively, the addition of Oa-POSS increases the deswelling rate of gels significantly. OP5-PN5 gels exhibit fast transparency change as a sign of phase transformation (Figure 7b); they can shrink and lose water to over 40 wt % within 10 min. The increased response rate may be attributed to several reasons. First, the hydrophobic Oa-POSS particle and its aggregates could form a microhydrophobic area inside the gel, which acts as a core to promote shrinking of the whole network. Second, small pores appear in the gel network due to the increased heterogeneity caused by Oa-POSS, as shown in Figure 6c, which is beneficial to the exclusion of water from pores. Moreover, as shown in OM images (Figure 7c,d), the surfaces of gels in the deswelling process show some small pores and wrinkles, while a similar phenomenon is not found in the surfaces of gels without POSS. This characteristic caused by the inhomogeneous shrinkage around the POSS particles also favors dehydration.

### 3.6. Structure Simulation of OPn-PNm Gels

**Figure 8:**
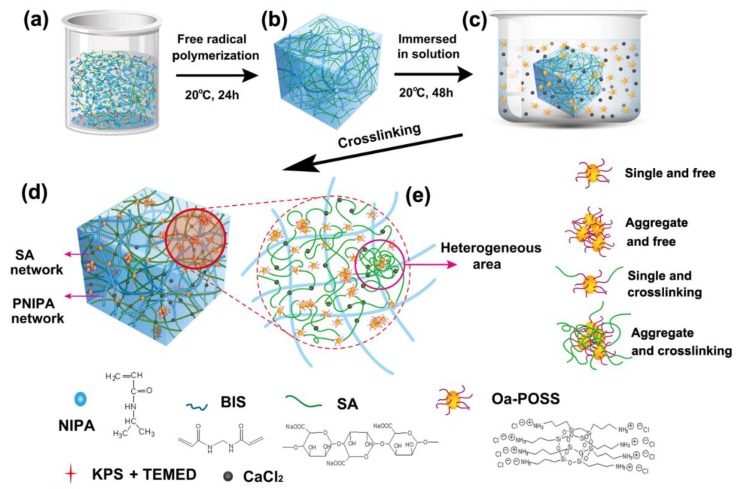
Schematic representation of synthesis process and structure model of OPn-PNm gels: (**a–d**) synthesis process of gels; (**e**) speculated microstructure of network, in which Oa-POSS particles have four states of existence: single and free (existing as single particle without cross-linking), aggregate and free (aggregate without cross-linking), single with cross-linking (single particle with cross-linking point, n = 1–4), aggregate and cross-linking (aggregate with cross-linking). The aggregate and free, and aggregate and cross-linking states cause heterogeneous areas inside the gels.

Based on the results obtained from comprehensive structure and performance characterization, the structure of the POSS hybrid IPN hydrogels was simulated, as shown in Figure 8. Considering the introduction of Oa-POSS particles in polymerization, combined with the improved strength, elasticity, and tighter network structure observed in SEM images, we can expect that at least part of the Oa-POSS particles work as a cross-linker to link SA macromolecular chains, and another part is only additive in the matrix of IPN gels. Moreover, Oa-POSS has eight ionic groups, and if all of them take part in cross-linking through ion exchange, the increased network density and heterogeneous structure should lead to great nonuniformity inside the gels, and the gels should be opaque, which is inconsistent with the facts. Therefore, it could be reasonably speculated that only one to four ionic groups in each Oa-POSS macromolecule participate in ion exchange with SA, and other ionic groups are free or combine with the NH groups of the matrix by hydrogen bonding. Based on the X-ray analysis, considering that there are many small pores in the observed network morphology and surface images of POSS hybrid hydrogels (SEM and OM images), which corresponds to the inhomogeneity feature caused by POSS particles, it can be assumed that although the dispersion of Oa-POSS inside the gels is uniform on the whole, there is still slight aggregation.

### 3.7. Drug Load and Release of OPn-PDm Gels

**Figure 9:**
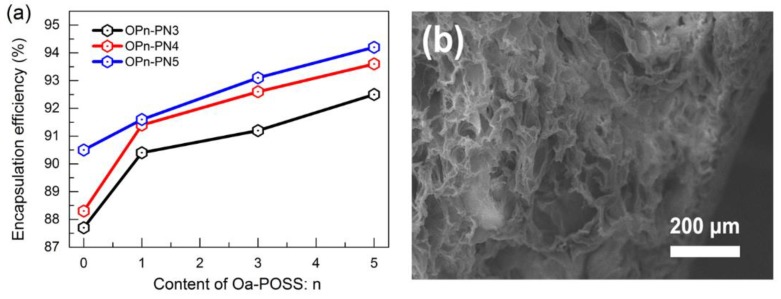
Drug-loading character of OPn-PNm gels: (**a**) drug loading content as a function of n and m for OPn-PNm gels; (**b**) SEM image of drug-loaded OP5-PN5 gels treated by freeze-drying.

In order to determine the application potential of these kinds of gels, a common drug-delivery macromolecule, bovine serum albumin (BSA), was used as a target to test drug loading and sustained release ability. Figure 9 exhibits the content of loaded drug in OPn-PNm gels as a function of POSS content. As shown in the figure, the interpenetrating network is quite beneficial to the drug loading, and the Oa-POSS also has a positive effect on drug loading. The micrograph of drug-loaded gels (Figure 9b) shows that the network of gels is filled with the drug. BSA has some amino acid groups that are easy to combine with the amino groups in NIPA and Oa-POSS. Moreover, the hydrophobic chains in drug macromolecules are inclined to combine with the hydrophobic POSS cage. Therefore, for drug loading, establishing the IPN structure and introducing POSS particles are effective in improving the loading ability.

**Figure 10:**
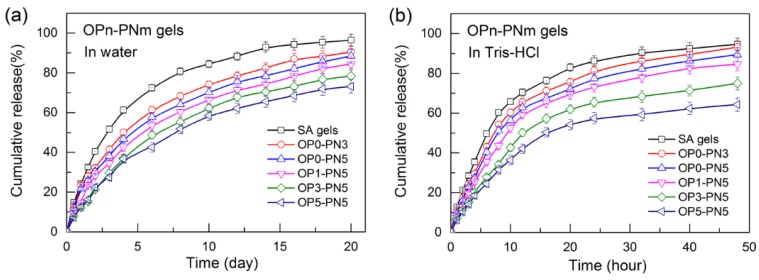
Release profiles of bovine serum albumin (BSA) in OPn-PNm gels in different solutions: (**a**) deionized water; (**b**) Tris-HCl buffer solution, 25 mM, pH = 7.3.

The profiles of drug release for SA gels and OPn-PNm gels are shown in Figure 10. As shown in Figure 10a, in pure water, all gels show a burst release of BSA initially for about 5 d, then the drug releases sequentially from the matrix within the studied period of 20 d, indicating the effective sustained release of drug. Moreover, a better release effect was found with increased PNIPA network and Oa-POSS content. The reason for this is the same as that for the better drug loading. When the release condition was changed to Tris-HCl buffer solution (25 mM, pH = 7.3), as shown in Figure 10b, although the total release character and rule are similar to those in pure water, the release rate of all gels becomes faster and the burst release phenomenon is weakened in POSS hybrid gels. Tris buffer solution is good for solving the protein, which accelerates the dissolution of BSA out of the gels. The POSS particles have better combination ability with BSA, resulting in a weaker burst release. Although the loading mass of OPn-PNm gels with high n and m is greater than that of pure SA gels, the release rate is still slower, showing a better sustained release effect.

It needs to be pointed out that the drug loading and release exploration here is only one specific example to display the potential of this gel as a drug-release system. As we know, different drugs with special molecular structure would have different interactions with the gel matrix, which would to be analyzed individually. For the OPn-PNm gels, the introduction of IPN structure and Oa-POSS could adjust the hydrophilic–hydrophobic character of the gels and improve their ability to be combined with certain drugs. On the other hand, the temperature responsiveness of this gel could also be applied for drug delivery in vitro. Correlative experiments were carried out in a simulated human body environment by using the micromolecule drug flutamide as the target drug. It was found that, compared to pure PNIPA gels, OPn-PNm gels had better drug loading and sustained release ability.

## 4. Conclusions

In this work, to develop novel biomass-based stimulus-responsive hydrogels with excellent properties, double network POSS hybrid hydrogels (named OPn-PNm gels) were synthesized by using Oa-POSS cationic particles to partly substitute CaCl_2_ as the co-cross-linking agent of SA and introducing a temperature-responsive PNIPA network into the SA hydrogels simultaneously, to obtain a series of transparent and homogeneous gels without obvious phase separation or aggregates. Through comprehensive analysis of the gels by FTIR, X-ray, TGA, DSC, SEM, and property characterization, it could be confirmed that the Oa-POSS was introduced into the gels successfully, and its content in the gels was determined by the concentration of POSS in a reaction in aqueous solution. Moreover, the addition of a second PNIPA network and Oa-POSS had a significant effect on the properties of gels, and several main conclusions are obtained.

First, the mechanical properties of OPn-PNm gels showed great change, which depended on the content of Oa-POSS and PNIPA network inside the gels. With the increase of PNIPA content, the strength and elongation at breakage of gels increased considerably, while the modulus was affected slightly. This is attributed to the stress loading mode of both networks: the rigid SA network as the main stress loader determines the modulus in initial deformation, the second PNIPA network helps to share more stress and keep the gels unbroken in bigger deformation. The increased Oa-POSS improved the strength and modulus of gels significantly, while the elongation at breakage of gels exhibited a considerable decrease. This is ascribed to the stress concentration caused by the increased heterogeneity of gels due to microaggregation of Oa-POSS particles. In addition, the compression resistance and resilience of gels were improved significantly by adding the second network and Oa-POSS, revealing that the network became stiffer. This network characteristic could also be proved by the rheological measurement results. Under the same frequency region, with the increase of the second PNIPA network and Oa-POSS content, the storage modulus and loss modulus of gels increased at different levels, indicating a more elastic network and stronger viscous behavior. Also, the damping effect of OPn-PNm gels was enhanced with the increased PNIPA network content. There was a stronger interface between the two polymer networks, resulting in greater energy consumption.

Second, through the research related to the swelling and deswelling properties of gels, it was found that the PNIPA network improved the swelling ability of gels significantly due to its hydrophilic nature, while Oa-POSS had an inverse effect due to the increased hydrophobicity, which could be supported by the results of water contact angle tests. The temperature responsiveness became obvious with the increase of PNIPA content. The VPTT of OPn-PNm gels was close to that of PNIPA gels and should shift to lower temperature with the increase of Oa-POSS content. However, the complete shrinking of OPn-PNm gels at high temperature was inhibited by the robust and hydrophilic SA network. Moreover, the OPn-PNm gels exhibited good swelling–deswelling repeatability under low–high temperature circulation due to the resilience of the gel network.

Third, the deswelling rate of OPn-PNm gels increased with the increased PNIPA and Oa-POSS content, the PNIPA network was the main driving force causing the shrinking of the network, and the Si–O skeleton of Oa-POSS acted as a hydrophobic core to accelerate the shrinking of polymer chains. Moreover, the introduction of Oa-POSS particles created some small pores inside the gel network, and the inhomogeneous shrinkage around POSS also formed small pores in the gel surface, all of which improved the deswellling rate.

Finally, to investigate the potential of these hybrid gels as a drug-release system, BSA was used as a target drug to analyze the drug-loading and -release properties of the OPn-PNm gels. As the results showed, the addition of PNIPA network and Oa-POSS particles could improve the drug-loading ability effectively due to their strong interaction with drug macromolecules. Although the burst release of drug still existed for all gels, the increased Oa-POSS content significantly improved the sustained release effect of gels in both water and Tris-HCl buffer solution.
